# Spatiotemporal control of axillary meristem formation by interacting transcriptional regulators

**DOI:** 10.1242/dev.158352

**Published:** 2018-12-10

**Authors:** Cui Zhang, Jin Wang, Stephan Wenkel, John W. Chandler, Wolfgang Werr, Yuling Jiao

**Affiliations:** 1 State Key Laboratory of Plant Genomics, Institute of Genetics and Developmental Biology, Chinese Academy of Sciences, and National Center for Plant Gene Research, Beijing 100101, China; 2 University of Chinese Academy of Sciences, Beijing 100049, China; 3 Copenhagen Plant Science Centre, University of Copenhagen, Thorvaldsensvej 40, 1871 Frederiksberg C, Copenhagen, Denmark; 4 Institute of Developmental Biology, Cologne Biocenter, University of Cologne, Zuelpicher Strasse 47b, D-50674 Cologne, Germany

**Keywords:** Axillary meristem, Branching, Stem cell, Transcription, *Arabidopsis*

## Abstract

Branching is a common feature of plant development. In seed plants, axillary meristems (AMs) initiate in leaf axils to enable lateral shoot branching. AM initiation requires a high level of expression of the meristem marker *SHOOT MERISTEMLESS* (*STM*) in the leaf axil. Here, we show that modules of interacting transcriptional regulators control *STM* expression and AM initiation. Two redundant AP2-type transcription factors, *DORNRÖSCHEN* (*DRN*) and *DORNRÖSCHEN-LIKE* (*DRNL*), control AM initiation by regulating *STM* expression. DRN and DRNL directly upregulate *STM* expression in leaf axil meristematic cells, as does another transcription factor, REVOLUTA (REV). The activation of *STM* expression by DRN/DRNL depends on REV, and vice versa. DRN/DRNL and REV have overlapping expression patterns and protein interactions in the leaf axil, which are required for the upregulation of *STM* expression. Furthermore, LITTLE ZIPPER3, another REV-interacting protein, is expressed in the leaf axil and interferes with the DRN/DRNL-REV interaction to negatively modulate *STM* expression. Our results support a model in which interacting transcriptional regulators fine-tune the expression of *STM* to precisely regulate AM initiation. Thus, shoot branching recruits the same conserved protein complexes used in embryogenesis and leaf polarity patterning.

## Abstract (precis)

**Summary:** Shoot branching uses interacting transcriptional regulators to fine-tune the spatiotemporal expression of *STM* and, thus, to precisely regulate axillary meristem initiation.

## INTRODUCTION

In contrast to animals, plants are sessile organisms with an enormous developmental plasticity to adapt to the changing environment. To this end, plants have innovated a branching growth habit (Coudert et al., 2015). In seed plants, shoot branches arise from axillary meristems (AMs, also termed lateral meristems) in, or near, the leaf axils (Schmitz and Theres, 2005; Wang and Jiao, 2018). Axillary bud development comprises two stages: initiation in the leaf axil and subsequent outgrowth or dormancy. The final pattern of branches to a large extent determines the architecture of the shoot system.

Recent studies have shown that AMs initiate from a group of leaf axil cells that constitute a meristematic cell lineage (Burian et al., 2016; Shi et al., 2016). These meristematic cells continuously express the meristem marker *SHOOT MERISTEMLESS* (*STM*). Whereas a low level of *STM* expression maintains meristematic competence, high levels of expression lead to AM initiation (Greb et al., 2003; Long and Barton, 2000; Shi et al., 2016). The maintenance of a low level of *STM* expression requires a low auxin concentration and response (Wang et al., 2014a,b). Before the formation of axillary buds, REVOLUTA (REV) upregulates *STM* expression to promote AM initiation (Shi et al., 2016). Subsequently, cytokinin activates *WUSCHEL* expression *de novo* to establish the AM (Wang et al., 2017). In addition, genetic studies have identified several transcription factor-encoding genes that regulate AM initiation in *Arabidopsis*, including *LATERAL SUPPRESSOR* (*LAS*), *REGULATOR OF AXILLARY MERISTEMS* (*RAX*), *CUP-SHAPED COTYLEDON* (*CUC*) and *REGULATOR OF AXILLARY MERISTEM FORMATION* (*ROX*) (Greb et al., 2003; Hibara et al., 2006; Müller et al., 2006; Raman et al., 2008; Yang et al., 2012). Genetic and molecular studies have revealed direct and indirect interactions among these genes to form a regulatory network (Raman et al., 2008; Tian et al., 2014).

REV belongs to the class III homeodomain-leucine zipper (HD-ZIPIII) family of transcription factors and plays pleiotropic roles in embryo, meristem, leaf and vascular development, including AM initiation (Brandt et al., 2012; Emery et al., 2003; Huang et al., 2014; Otsuga et al., 2001; Prigge et al., 2005; Talbert et al., 1995; Zhong and Ye, 1999). During leaf polarity patterning, the activity of REV and related HD-ZIPIII proteins is inhibited by microRNAs 165/166 (Emery et al., 2003; Mallory et al., 2004), and by interacting LITTLE ZIPPER (ZPR)-type microProteins (Kim et al., 2008; Wenkel et al., 2007).

The AP2 family transcription factors *DORNRÖSCHEN* (*DRN*), also named *ENHANCER OF SHOOT REGENERATION 1* (*ESR1*), and the related *DORNRÖSCHEN-LIKE* (*DRNL*/*ESR2*) also function in embryonic meristem and lateral organ development (Banno et al., 2001; Capua and Eshed, 2017; Chandler et al., 2007; Cole et al., 2009; Ikeda et al., 2006; Kirch et al., 2003; Nag et al., 2007). During embryogenesis, both DRN and DRNL heterodimerize with HD-ZIPIII proteins (Chandler et al., 2007). We have recently shown that AM initiation is compromised in the *drn-1* mutant (Tian et al., 2014), highlighting a novel function of DRN.

In this study, we show that DRN and DRNL redundantly promote AM initiation during the vegetative phase and show that DRN/DRNL and REV coordinately upregulate *STM* transcription in mature leaf axils. In early leaf development, ZPR3 is strongly expressed and may destabilize the DRN/DRNL-REV interaction in leaf axils, resulting in a low level of *STM* expression. These findings emphasize the dynamic interaction of transcriptional regulators as a core feature of developmental control. In addition, we show that the same DRN/DRNL-REV and ZRP3-REV interactions are shared by AM initiation, embryo development and leaf patterning, although with different downstream targets.

## RESULTS

### DRN and DRNL redundantly control axillary bud formation

**Fig. 1.**
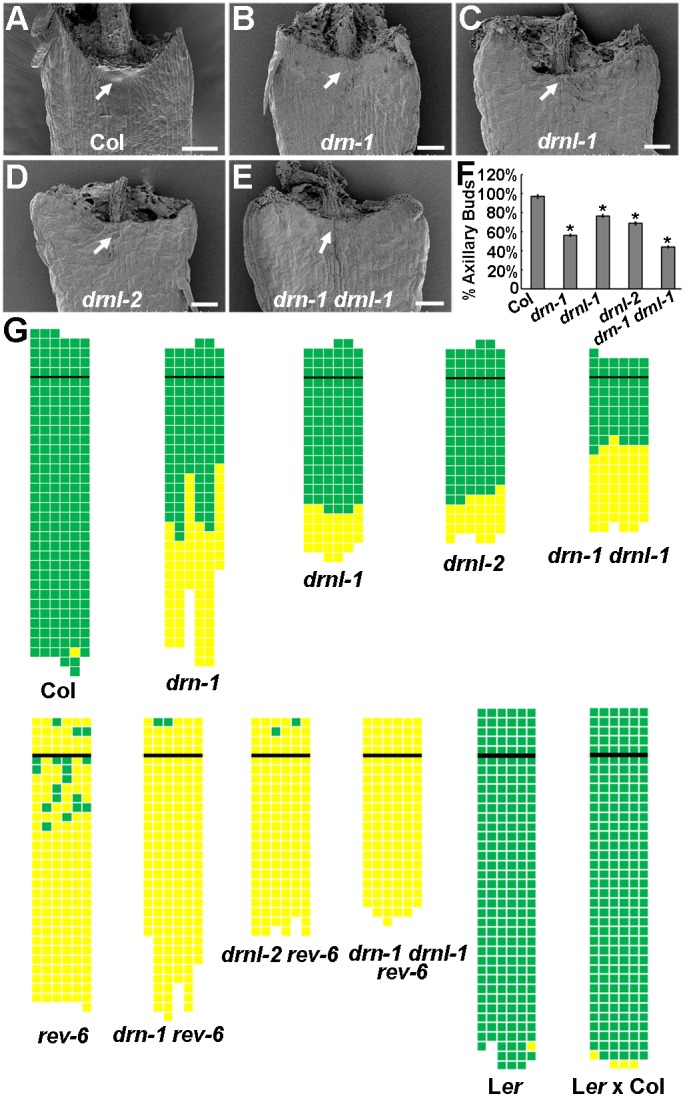
***DRN* and *DRNL* are required for AM initiation.** (A-E) Scanning electron micrographs of P_15_ rosette leaf axils in Col-0 wild type with a developing AM (dense cell mass, arrow) (A), and in *drn-1* (B), *drnl-1* (C), *drnl-2* (D) and *drn-1 drnl-1* (E) mutants with bare axils (arrows). (F) Axillary bud formation in *drn-1*, *drnl-1*, *drnl-2* and *drn-1 drnl-1* mutants during vegetative development in comparison with Col-0 wild-type plants. The percentage values indicate the mean proportion of axillary buds formed over the total number of leaves along the shoot axis (plants analyzed >20). Error bars indicate the s.d. *P<0.01 between wild type and each mutant. (G) Schematic representation of axillary bud formation in leaf axils of Col-0 wild-type plants; *drn-1*, *drnl-1*, *drnl-2*, *drn-1 drnl-1*, *rev-6*, *drn-1 rev-6*, *drnl-2 rev-6* and *drn-1 drnl-1 rev-6* mutant plants; L*er*, and mixed L*er* and Col-0 ecotypes. The thick black horizontal line represents the border between the youngest rosette leaf and the oldest cauline leaf. Each column represents a single plant, and each square within a column represents an individual leaf axil. The bottom row represents the oldest rosette leaf axils, with progressively younger leaves above. Green indicates the presence of an axillary bud; yellow indicates the absence of an axillary bud in any particular leaf axil. Scale bars: 100 μm.

We have recently shown via a genome-wide study that *DRN* is required for AM initiation (Tian et al., 2014). This current study aimed to understand in more detail how *DRN* regulates the formation of AMs in rosette leaf axils in the vegetative shoots. First, we tested whether the *DRN* paralogue *DRNL* is also required for this process. In wild-type *Arabidopsis* plants, the first visible evidence of AM formation by scanning electron microscopy was a cluster of small and proliferating cells at the adaxial leaf base (Fig. 1A). However, these proliferating cell clusters were absent in most early rosette leaf axils in *drn-1*, *drnl-1*, *drnl-2* and *drn-1 drnl-1* mutants (Fig. 1B-E). The absence of expression of the corresponding genes was confirmed (Fig. S1). In wild-type plants grown in short days, axillary buds develop from each rosette leaf, except from the two cotyledons and some of the first-formed true leaves (Fig. 1F,G). In contrast, *drn* and *drnl* mutants flower earlier and show a strong reduction in axillary bud formation, especially in rosette leaves formed in the early and mid-phase of vegetative development (Fig. 1F,G). The *drn-1 drnl-1* double mutant shows more serious defects in axillary bud formation than either *drn* or *drnl* single mutant (Fig. 1F,G), suggesting that *DRN* and *DRNL* have important redundant functions in AM initiation.

### *DRN*/*DRNL* and *REV* genetically co-regulate AM initiation

During embryogenesis, REV can dimerize with DRN/DRNL (Chandler et al., 2007). To test whether DRN/DRNL-REV heterodimers are also recruited during AM initiation, we sought genetic evidence by constructing *drn-1 rev-6*, *drnl-2 rev-6* and *drn-1 drnl-1 rev-6* double and triple mutants. Although short-day-grown *rev-6* loss-of-function plants showed a strong reduction in axillary bud formation (Otsuga et al., 2001; Talbert et al., 1995), axillary buds occasionally formed in the axils of cauline leaves and of rosette leaves formed during late vegetative development. The *drn-1 rev-6* and *drnl-2 rev-6* mutants showed further reductions in axillary bud formation. Additionally, the defect in axillary bud formation in the *drn-1 drnl-1 rev-6* mutant was almost completely penetrant (Fig. 1G). This increased phenotypic penetrance in double and triple mutant plants compared with *rev* single mutants suggests that *DRN*/*DRNL* and *REV* have combinatorially important functions that converge to form axillary meristems, but that they might also provide individual contributions. It has been proposed that AM initiation during vegetative and reproductive stages requires different sets of genes (Hempel and Feldman, 1994; Huang et al., 2012; Yang and Jiao, 2016). Compared with early rosette leaves, the AM defect becomes less severe in axils of later-initiated rosette leaves and cauline leaves in *drn*, *drnl* and *drn drnl* mutants. Expression of *pREV::REV-GR-HA* could not complement the *drn-1 drnl-1* AM defect from early rosette leaf axils, but resulted in slightly more AMs in late rosette leaves and cauline leaves (Fig. S2A)*.* Consistently, *p35S::DRN-GR* could not complement the *rev-6* AM defect from early rosette leaf axils (Fig. S2B)*.* Thus, DRN and DRNL preferentially affect the AM initiation pathway during early vegetative stages, more than during later stages and reproduction, which affects cauline leaves and later-initiated rosette leaves.

### Expression patterns of *DRN* and *DRNL* in leaf axils

**Fig. 2.**
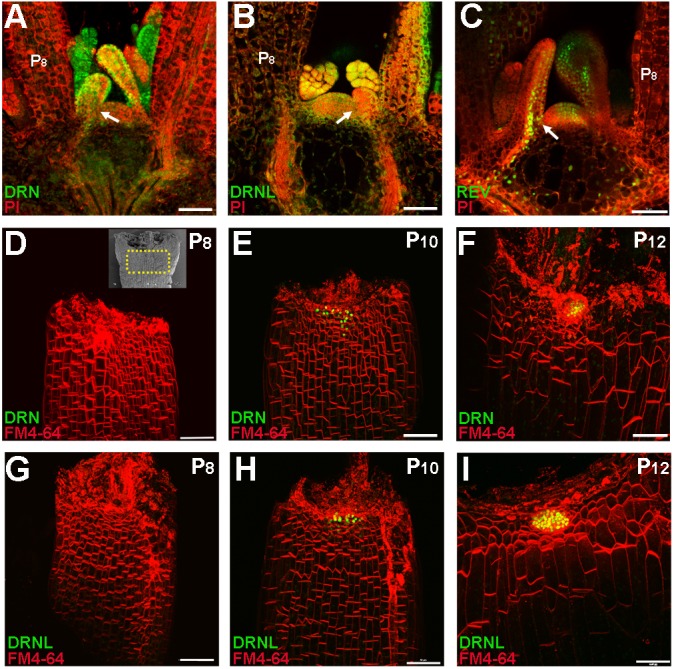
***DRN* and *DRNL* are expressed in leaf primordia and accumulate in the leaf axil prior to AM initiation.** (A-C) Expression of *pDRN::DRN-GFP* (A), *pDRNL::DRNL-CFP* (B) and *pREV::REV-Venus* (C) in the vegetative shoot apex and leaf primordia. Longitudinal sections of 14-day-old plant shoot apices were stained with propidium iodide (PI, red); fluorescent signals are shown in green. Arrows indicate leaf axils. Fluorescent signals are present in the leaf axils. (D-I) Reconstructed view of the epidermal layer of P_8_ (D,G), P_10_ (E,H) and P_12_ (F,I) leaf axils with *pDRN::DRN-GFP* (D-F) or *pDRNL::DRNL-CFP* (G-I) expression in green and FM4-64 staining in red showing the location of AM progenitor cells. The inset in D shows a scanning election micrograph of a rosette leaf axil at a similar stage; the region within the yellow dotted box roughly corresponds to the imaged regions shown in D-I. All leaves were removed from 17-day-old plants. Note the enrichment of *DRN-GFP* and *DRNL-CFP* signals in P_10_ and P_12_ leaf axils. Scale bars: 50 μm.

Because AMs initiate from organ boundary cells located at the adaxial side of leaf axils, we investigated whether *DRN* and *DRNL* are expressed in the leaf axils. We initially used *pDRN::GUS* and *pDRNL::GUS* reporter lines that recapitulate RNA *in situ* hybridization patterns (Kirch et al., 2003; Nag et al., 2007), and detected GUS activity that expanded throughout the shoot apex and young primordia for both *pDRN::GUS* and *pDRNL::GUS* (Fig. S3). We subsequently analyzed the expression patterns of the *pDRN::DRN-GFP* and *pDRNL::DRNL-CFP* reporters (Chandler et al., 2011; Cole et al., 2009) in young leaf primordia, which were comparable with those of RNA *in situ* hybridization (Fig. 2A,B). We observed broad *pDRN::DRN-GFP* signals in leaf primordia, including boundary cells (Fig. 2A, Fig. S4C). In addition, DRN was very strongly expressed in AMs (Fig. S4C). A similar pattern of *pDRNL::DRNL-CFP* expression was detected in primordia, boundary cells and AMs (Fig. 2B, Fig. S4D). Similar to *DRN* and *DRNL*, *REV* was also expressed in leaf axils (Fig. 2C) and this expression was relatively low in leaves after P_8_ (Shi et al., 2016). In contrast to the expression of *DRN* and *DRNL*, expression of *REV* was more adaxial specific (Otsuga et al., 2001).

We next analyzed *DRN* and *DRNL* expression in older leaf axils. Imaging of *pDRN::DRN-GFP* in leaves showed that GFP signal was absent from the adaxial side of the leaf axil between P_8_ and P_9_ stages (Fig. 2D, Fig. S4A). Starting from P_10_, the GFP signal became restricted to the center of the leaf axils (Fig. 2E,F, Fig. S4B), which is the site of presumptive AM initiation. We also observed similar dynamic expression of *pDRNL::DRNL-CFP* in leaf axils (Fig. 2G-I). The expression of *REV* was similarly restricted to the center of leaf axils in P_9_ and older leaves (Shi et al., 2016). The enrichment of *DRN*, *DRNL* and *REV* expression in leaf axils is consistent with the upregulation of *STM* expression in P_10_ and older leaves, which is crucial for AM initiation (Shi et al., 2016).

### *DRN* and *DRNL* regulate *STM* expression

**Fig. 3.**
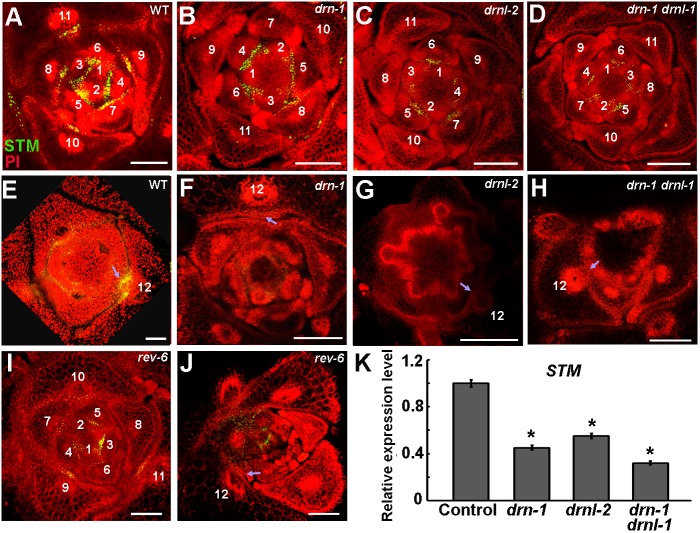
**Attenuated *STM* expression in *drn* and *drnl* mutants.** (A-D) Patterns of *pSTM::STM-Venus* (green) in transverse sections through the vegetative shoot apex of 28-day-old wild-type Col-0 (A), *drn-1* (B), *drnl-2* (C) and *drn-1 drnl-1* (D) plants. Plants are stained with propidium iodide (PI, red). The *STM* expression level is decreased in the mutants compared with wild type. (E-H) Patterns of *pSTM::STM-Venus* expression in P_12_ leaf axils in wild-type Col-0 (E), *drn-1* (F), *drnl-2* (G) and *drn-1 drnl-1* (H). *STM* expression levels are lower in mature leaves in the mutants than in wild type. (I,J) *STM-Venus* expression levels in *rev-6* in young (I) and mature (J) leaves. Arrows indicate leaf axils; numbers in A-J indicate leaf stages. (K) RT-qPCR analysis indicates that *STM* expression is significantly reduced in *drn-1*, *drnl-2* and *drn-1 drnl-1* mutant plants. Vegetative shoots with the leaves removed were analyzed. Error bars indicate s.d. *P<0.01 (Student's *t*-test). Scale bars: 100 μm.

AM initiation requires cells that continuously express *STM*. A low level of *STM* expression maintains meristematic competence, whereas high *STM* expression leads to AM initiation (Greb et al., 2003; Long and Barton, 2000; Shi et al., 2016). In wild-type plants, the expression of *STM* persists in young leaf axils (Fig. 3A). We found that *STM* expression was maintained in *drn-1*, *drnl-2* and *drn-1 drnl-1* mutants, but was much lower than in wild-type plants (Fig. 3B-D, Fig. S5). In wild type, the number of *STM*-expressing cells and the level of *STM* expression increased from the P_11_ stage, just prior to the stage at which AMs become morphologically visible (Fig. 3E) (Shi et al., 2016). However, *STM* was not upregulated in the mutants during leaf maturation (Fig. 3F-H, Fig. S5). The level of *STM* expression in *drn* and *drnl* mutants was similar to that in the *rev-6* mutant (Shi et al., 2016) (Fig. 3I,J). Reverse transcription quantitative PCR (RT-qPCR) analysis also confirmed the upregulation of *STM* by *DRN* and *DRNL*. The level of *STM* expression in shoot apices that are enriched with leaf axils by leaf removal, was significantly reduced in *drn-1*, *drnl-2* and *drn-1 drnl-1* plants compared with wild-type plants (Fig. 3K). This low level of *STM* expression in leaf axils is insufficient for AM initiation (Shi et al., 2016), and thus explains the AM initiation defects in *drn* and *drnl* mutants.

**Fig. 4.**
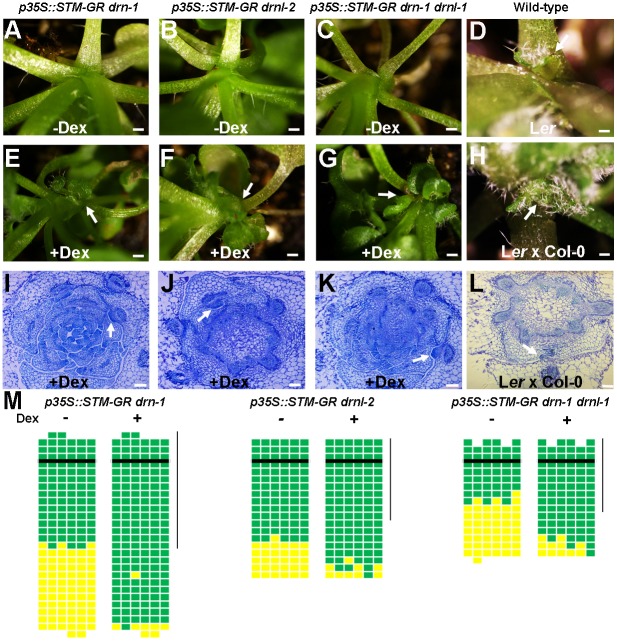
**Overexpression of *STM* rescues axillary bud deficiency in *drn* and *drnl* mutants.** (A-C) Higher magnification of rosette leaf axils in mock-treated *p35S::STM-GR drn-1* (A), *p35S::STM-GR drnl-2* (B) and *p35S::STM-GR drn-1 drnl-1* (C) plants showing the absence of an axillary bud. (E-G) Higher magnification of rosette leaf axils in Dex-treated *p35S::STM-GR drn-1* (E), *p35S::STM-GR drnl-2* (F) and *p35S::STM-GR drn-1 drnl-1* (G) plants showing the presence of axillary buds (arrows). (D,H) Higher magnification of rosette leaf axils in mock-treated L*er* and mixed L*er*×Col-0 ecotypes showing the presence of axillary buds (arrows). (I-L) Transverse sections through vegetative shoot apices of 28-day-old Dex-treated *p35S::STM-GR drn-1* (I), *p35S::STM-GR drnl-2* (J), *p35S::STM-GR drn-1 drnl-1* (K) and mixed L*er*×Col-0 ecotypes (L) stained with Toluidine Blue O, showing the presence of axillary buds (arrows) in rosette leaf axils. (M) Schematic representation of axillary buds in leaf axils with or without Dex induction. Green indicates the presence of an axillary bud; yellow indicates the absence of an axillary bud. Plants were grown under short-day conditions for 15 days without treatment; leaf axil regions were treated with 10 µM Dex every second day for another 15 days and then transferred to long-day conditions without treatment until axillary buds were counted. The vertical line indicates leaves initiated during Dex treatment. Horizontal lines indicate the border between the youngest rosette leaf and the oldest cauline leaf. Scale bars: 2 cm in A-H; 100 μm in I-L.

Previous studies have shown that ectopic *STM* activity can induce meristems from undifferentiated (and presumably meristematic) cells, but not differentiated cells (Brand et al., 2002; Shi et al., 2016). To test whether leaf axil cells in *drn* and *drnl* mutants remain meristematic, i.e. are competent to respond to *STM* activity, we introduced *p35S::STM-GR* into *drn-1*, *drnl-2* and *drn-1 drnl-1* plants. In these plants, dexamethasone (Dex) can induce the nuclear translocation of a STM-glucocorticoid-receptor (GR) fusion protein to activate STM function. We found that axillary buds could be induced from young and mature leaf axils in these mutants by Dex application, although at a slightly lower frequency in the *drn-1 drnl-1* double mutant (Fig. 4). REV also upregulates *STM* expression to promote AM initiation. Induced *STM* expression can similarly complement the *rev* mutant phenotype in bud formation (Shi et al., 2016). Genetically, *STM* overexpression also complemented branch suppression in the *drn-1 drnl-1 rev-6* triple mutant (Fig. S6). Thus, leaf axil cells in *drn-1*, *drnl-2* and *drn-1 drnl-1* that express *STM* at a low level remain meristematic, as observed in *rev-6* (Shi et al., 2016).

### DRN and DRNL directly activate *STM* expression

**Fig. 5.**
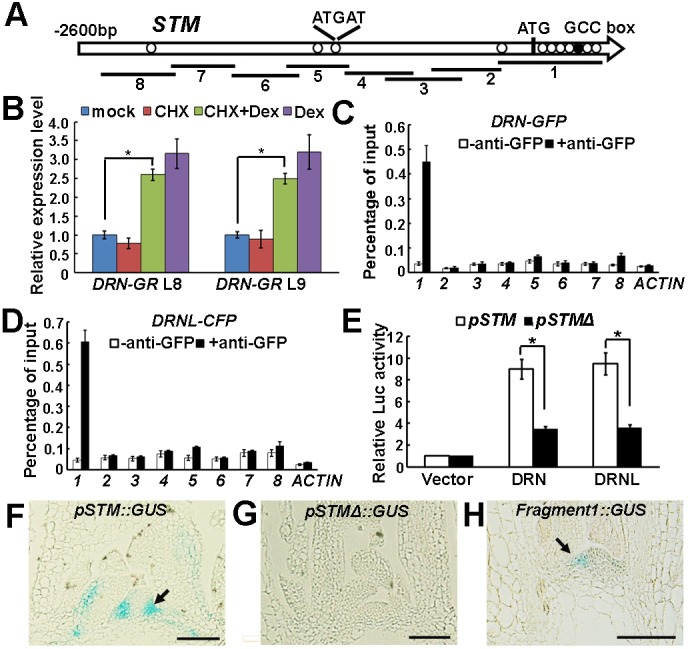
**DRN and DRNL regulate *STM* expression via binding to a conserved promoter motif.** (A) Schematic representation of the *STM* genomic region. The black circle and white circles indicate the GCCGCC motif and the ATGAT motif, respectively; ATG denotes the translation start site. Eight PCR fragments were designed for ChIP analysis. (B) RT-qPCR analysis of *STM* expression using *p35S::DRN-GR* vegetative shoots (with the leaves removed) before and after simultaneous Dex and CHX treatment for 2 h. The vertical axis indicates the relative mRNA amount compared with the amount in the mock treatment. Error bars indicate the s.d. Two independent transgenic lines were used. *P<0.01 (Student's *t*-test). (C,D) ChIP-qPCR analysis indicates binding of DRN-GFP (C) and DRNL-CFP (D) to fragment 1. Error bars indicate the s.d. (E) Relative *Luc* reporter gene expression in transcriptional activity assays in *Arabidopsis* protoplasts. The 3.0 kb *STM* promoter region (*pSTM*) or the same region without fragment 1 (as indicated in A, *pSTMΔ*) was co-transformed with *p35S::DRN* or *p35S::DRNL*, and *p35S::GUS* was the internal control. Data are mean±s.d. for three independent biological experiments, each performed in triplicate. *P<0.01 (Student's *t*-test). (F-H) Pattern of GUS expression driven by *pSTM* (F), *pSTMΔ* (G) and fragment 1 (H) in longitudinal sections through a vegetative shoot apex of 30-day-old plants. To compare signals, plants were stained in parallel for 6 h, and sections were placed on the same slides for detection. The GUS signal is barely detectable in leaf axils of *pSTMΔ::GUS* plants (F) but weakly detectable in *fragment1::GUS* (H). Arrows highlight leaf axils. See Fig. S7 for more examples. Scale bars: 100 μm in F-H.

To test whether DRN can directly activate *STM* expression, we generated Dex-inducible *p35S::DRN-GR* lines and measured the effect of DRN activation on the expression of *STM* by RT-qPCR. DRN activation resulted in a rapid increase in *STM* mRNA levels within 2 h of treatment in the presence or absence of the protein synthesis inhibitor cycloheximide (CHX) (Fig. 5B). These results suggest that induction of *STM* does not require *de novo* protein synthesis and that *STM* is probably a direct target of DRN.

We next performed chromatin immunoprecipitation (ChIP) assays using leaf axil-enriched shoot tissues to examine whether DRN and DRNL directly bind to the *STM* promoter *in vivo*. We designed primers upstream of the start codon that covered the ∼2.6 kb region or spanned the start codon (Fig. 5A). Using antibodies against GFP, we found that DRN-GFP strongly associated with a region containing the start codon and a GCCGCC motif (GCC box), the conserved binding site for DRN and related AP2-type transcription factors (Banno et al., 2006), in vegetative-stage shoot apex tissue that was enriched for axils by leaf removal (Fig. 5C). Similarly, we found that DRNL-CFP could associate with the same region (Fig. 5D), which we term fragment 1. Fragment 1 also contains multiple ATGAT motifs, which are the conserved binding site for REV, and we recently demonstrated that REV also binds to fragment 1 (Shi et al., 2016).

A transient transfection assay in protoplasts further confirmed that DRN and DRNL could bind to *STM* genomic regions, especially fragment 1, and upregulate *STM* expression (Fig. 5E). Although both DRN and DRNL activated a 3.0 kb *STM* promoter-driven *Luciferase* reporter gene (*pSTM::Luc*), the activation of *pSTMΔ::Luc*, in which fragment 1 was replaced with a CaMV 35S minimal promoter (contains the TATA box), was substantially reduced.

To further confirm the importance of fragment 1 for *STM* expression, we constructed a GUS reporter (*pSTM::GUS*) containing 6.3 kb of the *STM* promoter upstream of the start codon, which also included fragment 1. We also constructed a *pSTMΔ::GUS* reporter, in which fragment 1 was replaced by a CaMV *35S* minimal promoter. From multiple independent transgenic lines (>5), we repeatedly found that *pSTM::GUS* expression recapitulated RNA *in situ* patterns, with strong expression in the shoot apex and leaf axils (Fig. 5F, Fig. S7A-D). By contrast, *pSTM*Δ::*GUS* plants showed barely detectable GUS signals in these tissues (Fig. 5G, Fig. S7E-H), which might be partly due to the inhibitory *cis*-element K-box and RB-box remaining in the promoter (Aguilar-Martínez et al., 2015; Uchida et al., 2007). To directly analyze the contribution of fragment 1 to *STM* expression, we also generated a *fragment 1::GUS* reporter, in which *GUS* expression remained detectable but was less enriched in the leaf axil compared with that using the 6.3 kb promoter (Fig. 5H, Fig. S7I-L). We therefore conclude that fragment 1, which is bound by DRN, DRNL and REV, is crucial for *STM* expression.

### DRN and DRNL interact with REV to activate *STM* expression

DRN, DRNL and REV can directly activate *STM* expression by binding to the same promoter region, and these transcription factors significantly overlap in expression. Similar to *rev* mutants, *drn* and *drnl* mutants have AM initiation defects. During embryogenesis, DRN/DRNL can physically interact with REV (Chandler et al., 2007). During AM initiation, *REV* is also expressed at progressively higher levels in the leaf axil (Shi et al., 2016), in a similar way to *DRN* and *DRNL*. We therefore speculate that the DRN/DRNL-REV interaction occurs during AM initiation, and is crucial for the activation of *STM* expression.

**Fig. 6.**
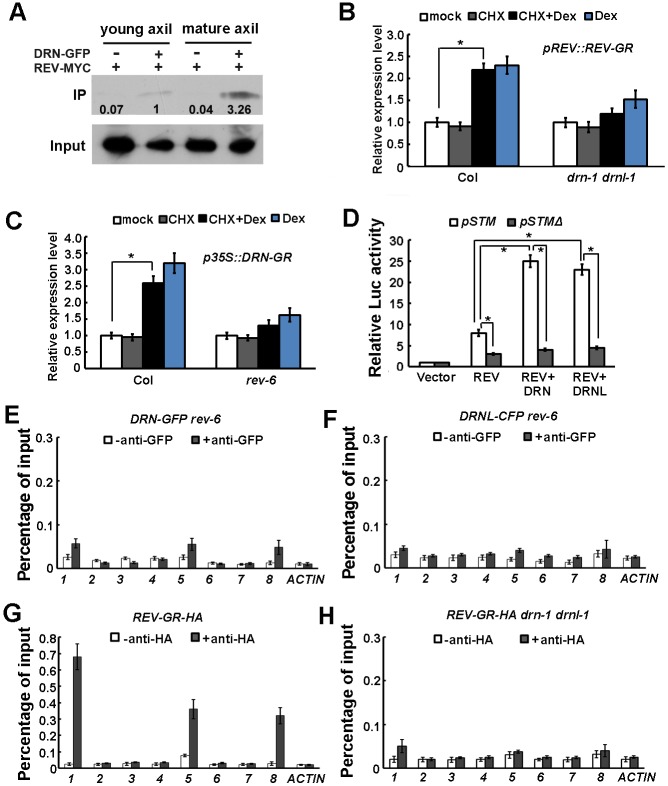
**DRN and DRNL enhance the activation of *STM* expression by REV.** (A) Co-IP assay indicating that DRN and REV interact *in vivo* and the interaction is stronger in mature leaf (older than P_10_) axils than in young leaf (younger than P_10_) axils. The numbers below the blots indicate the relative ratios of the signal intensity between IP and input bands (IP/input). The ratios were normalized to the IP band in young leaf axils of crossed marker lines. An anti-GFP antibody was used for IP and an anti-MYC antibody as a probe. Input shows the amount of DRN-GFP protein used in the IP assay. (B) RT-qPCR analysis of *STM* expression in *pREV::REV-GR* and *pREV::REV-GR drn-1 drnl-1* vegetative shoots (with the leaves removed) before and after simultaneous Dex and CHX treatment for 2 h. The vertical axis indicates the relative mRNA amount compared with the amount in the mock treated. Error bars indicate the s.d. *STM* activation is reduced in *pREV::REV-GR drn-1 drnl-1*. *P<0.01 (Student's *t*-test). (C) RT-qPCR analysis of *STM* expression in *p35S::DRN-GR* and *p35S::DRN-GR rev-6* vegetative shoots (with the leaves removed) before and after simultaneous Dex and CHX treatment for 2 h. The vertical axis indicates the relative mRNA amount compared with the amount in the mock treatment. Error bars indicate s.d. *STM* activation is reduced in *p35S::DRN-GR rev-6*. *P<0.01 (Student's *t*-test). (D) Relative *Luc* reporter gene expression in transcriptional activity assays in *Arabidopsis* protoplasts. The *pSTM::Luc* or the *pSTMΔ::Luc* constructs were co-transformed with *p35S::REV* alone, *p35S::REV* and *p35S::DRN*, or *p35S::REV* and *p35S::DRNL*; *p35S::GUS* was the internal control. Data are mean±s.d. Error bars are derived from three independent biological experiments, each performed in triplicate. Note the enhanced activation of *STM* expression by DRN and DRNL. *P<0.01 (Student's *t*-test). (E,F) ChIP-qPCR analysis demonstrates the reduced binding of DRN-GFP (E) and DRNL-CFP (F) to the *STM* genomic region (as in Fig. 5A) in *rev-6* plants. Compare binding with that in Fig. 5C,D for Col-0 wild-type plants. Error bars indicate the s.d. (G,H) ChIP-qPCR analysis demonstrates binding of REV-GR-HA to the *STM* genomic region in Col-0 wild-type plants (G); this is reduced in *drn-1 drnl-1* plants (H). Error bars indicate s.d.

To examine the interaction between DRN and REV during *in vivo* development, we performed co-immunoprecipitation (Co-IP) using *pDRN::DRN-GFP p35S::REV-MYC* plants. Using leaf axil-enriched shoot tissues, we detected an interaction between DRN-GFP and REV-MYC when immunoprecipitated with an anti-GFP antibody and probed with an anti-MYC antibody. Furthermore, using the same amount of DRN-GFP, we found that the band indicating the amount of (35S-driven) REV-MYC was about threefold stronger in old leaf axil-enriched tissues than in young leaf axil-enriched tissues (Fig. 6A).

To test whether the detected DRN/DRNL-REV interaction is required for the activation of *STM* expression, we introduced *pREV::REV-GR-HA* into the *drn-1 drnl-1* background and measured the effect of REV activation on *STM* expression, which was substantially reduced compared with that in wild-type siblings (Fig. 6B). Consistently, the effect of DRN activation on *STM* expression was also compromised in the *rev-6* background in comparison with that in wild-type siblings (Fig. 6C).

A transient transfection assay in protoplasts also confirmed that the DRN/DRNL-REV interaction can upregulate *STM* expression (Fig. 6D). Although transformation with only DRN, DRNL or REV resulted in the activation of *STM* expression (Fig. 5E, Fig. 6D), co-transformation of DRN and REV or DRNL and REV resulted in a ∼2.5-fold stronger activation of *STM* expression (Fig. 6D).

Furthermore, our results show that interaction between DRN/DRNL and REV is crucial for their binding to the *STM* promoter region. Using the same experimental set-up, we found that both DRN-GFP and DRNL-CFP showed reduced association with fragment 1 of the *STM* promoter region in the *rev-6* background than in wild-type plants (Fig. 6E,F, compare with Fig. 5C,D). Similarly, REV-GR-HA showed weaker binding to the *STM* promoter in the *drn-1 drnl-1* background than in wild-type siblings (compare Fig. 6G with H). These results indicate that the DRN/DRNL-REV interaction is recruited during AM initiation, and this interaction cooperatively promotes binding of the proteins to the *STM* genomic region. Taken together, our data demonstrate that DRN/DRNL and REV function as a complex to promote the expression of downstream target genes during AM initiation from early rosette leaf axils.

### ZPR3 destabilizes the DRN/DRNL-REV interaction

**Fig. 7.**
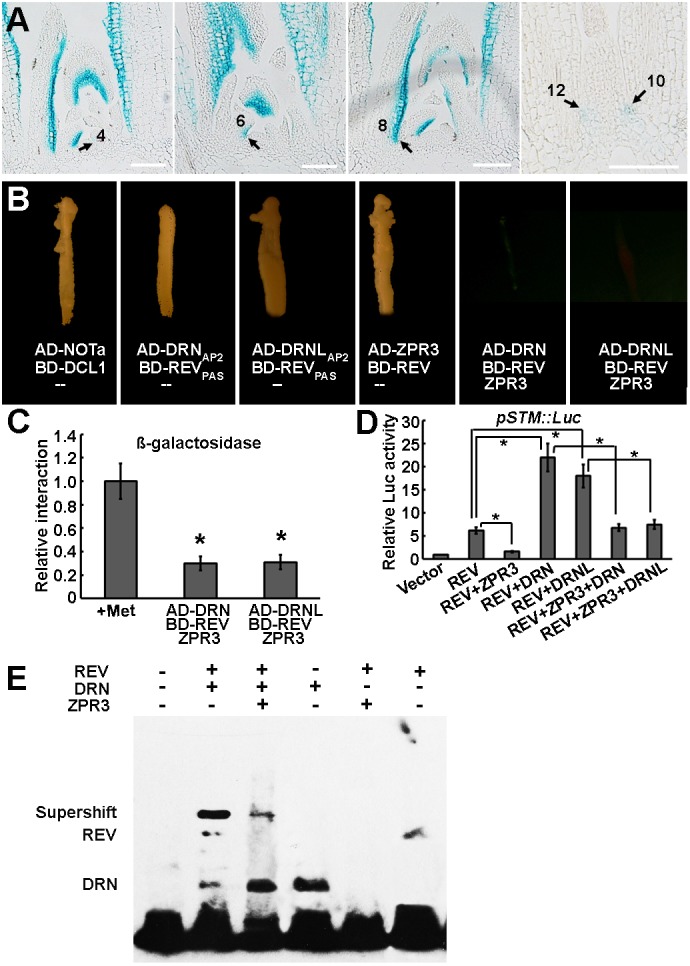
**ZPR3 interferes with the DRN/DRNL-REV interaction and inhibits *STM* expression.** (A) Patterns of *ZPR3*-promoter-driven GUS expression in serial longitudinal 8 μm sections through the vegetative shoot apex of a 30-day-old wild-type-like plant. Arrows indicate the leaf axils. The GUS signals are weaker in the leaf axils of P_10_ and older leaves. See Fig. S8D-G for additional transverse sections. Scale bars: 100 μm. (B) Y2H and Y3H assay showing the disruption of the DRN/DRNL-REV interaction by ZPR3. Yeast growth on SD-Leu-Trp-His-Ade plates showing that DRN, DRNL and ZPR3 interact with REV, respectively. The interaction of DRN or DRNL with REV was weakened after the induction of ZPR3 activity. AD-NOTa and BD-DCL1 were used as positive controls. (C) Relative β-galactosidase activity of the UAS-driven β-galactosidase reporter measured before and after ZPR3 induction in Y3H. Constructs and additional results are shown in Fig. S9. The data are mean values of three replicates±s.d. *P<0.01 (Student's *t*-test). (D) Relative *Luc* reporter gene expression in transcriptional activity assays in *Arabidopsis* protoplasts. The *pSTM::Luc* construct was co-transformed with *p35S::REV* alone, *p35S::REV*+*p35S::ZPR3*, *p35S::REV*+*p35S::DRN*, *p35S::REV*+*p35S::DRNL*, *p35S::REV*+*p35S::ZPR3*+*p35S::DRN* or *p35S::REV*+*p35S::ZPR3*+*p35S::DRNL*; *p35S::GUS* was the internal control. Data are mean±s.d. Error bars are derived from three independent biological experiments, each performed in triplicate. Note the suppression of REV activation and DRN/DRNL–REV co-activation of *STM* expression by ZPR3. *P<0.01 (Student's *t*-test). (E) Supershift in EMSA, indicating that REV and DRN interact and bind to a biotin-labeled *STM* promoter fragment. The addition of ZPR3 decreased the intensity of the supershift band of DRN and REV; 2 µg DRN and ZPR3, and 1 µg REV protein were used for incubation.

During leaf polarity patterning, REV can interact with ZPR proteins and this interaction inhibits REV function (Kim et al., 2008; Wenkel et al., 2007). We speculated that ZPR proteins might also participate in the regulation of AM initiation. To examine this, we first analyzed the tissue-specific expression pattern of *ZPR3* and *ZPR4* expressions and that of the related genes *ZPR1* and *ZPR2* using RT-qPCR in various organs. ZPR genes were more highly expressed in boundary-enriched tissues such as the shoot apex and flowers than in leaves (Fig. S8A,B). ZPR gene expression was analyzed at different stages of primordium development, and was higher in young leaves and decreased in mature leaves where AMs will initiate (Fig. S8C). Using a *pZPR3::GUS* reporter (Wenkel et al., 2007), we detected GUS activity in the adaxial domain of leaf primordia, including the leaf axils (Fig. 7A, Fig. S8D-H). Notably, GUS activity in the leaf axils was substantially reduced in P_10_ and older leaves. The reduction in *ZPR3* leaf axil expression during leaf maturation is consistent with the upregulation of *STM* expression beginning in P_11_, prior to the morphological appearance of axillary buds (Greb et al., 2003; Shi et al., 2016). Overexpression of *ZPR3* led to fewer axillary buds in the axils of cauline and rosette leaves in *p35S::ZPR3* transgenic lines (Fig. S8I-K).

We next tested whether the ZPR3 protein inhibits the DRN/DRNL-REV interaction to inhibit downstream gene expression, using the yeast three-hybrid (Y3H) assay. In yeast, the AP2 domain of DRN or DRNL fused to the activation domain of GAL4 (AD-DRN_AP2_ or AD-DRNL_AP2_) can interact with the PAS domain of REV fused to the binding domain of GAL4 (BD-REV_PAS_) (Fig. 7B), as previously reported (Chandler et al., 2007). Similar results were obtained using full-length DRN/DRNL and REV (Fig. S9). In addition, AD-ZPR3 interacted with BD-REV in yeast, as shown previously (Kim et al., 2008; Wenkel et al., 2007). The interaction between AD-DRN or AD-DRNL and BD-REV was reduced in the presence of ZPR3 (Fig. 7B,C). Similarly, the introduction of DRN or DRNL in the Y3H assay interfered with the REV-ZPR3 interaction (Fig. S9). To confirm that DRN/DRNL competes with ZPR3 to interact with REV, we also introduced DRN or DRNL without the REV-interacting AP2 domain (Chandler et al., 2007). The truncated DRNΔAP2 and DRNLΔAP2 versions were no longer able to disrupt the ZPR3-REV interaction (Fig. S9).

Using transient transfection assays in protoplasts, we not only confirmed the competition between DRN/DRNL-REV and ZPR3-REV interactions, but also showed that ZPR3 inhibits the DRN/DRNL-REV-mediated activation of *STM* expression. When ZPR3 was co-transformed into protoplasts with REV, the activation of *STM* was substantially reduced, leading to significantly reduced *Luc* expression (Fig. 7D). However, co-transformation of DRN/DRNL, ZPR3 and REV fully restored *Luc* expression to a higher level but still much less than that following REV-DRN or REV-DRNL co-transformation (Fig. 7D, and compare with Fig. 6D). To analyze the effect of DRN-REV interaction on DNA-binding activity, we performed an electrophoretic mobility shift assay (EMSA) using biotin-labeled fragment 1 of the *STM* promoter. The addition of both DRN and REV resulted in a lower mobility than the addition of either single protein, suggesting supershift protein-DNA binding by heterodimerization. ZPR3 decreased the intensity of the supershift band of DRN and REV (Fig. 7E), suggesting that ZPR3 interferes with the interaction between the DRN-REV complex and the *STM* promoter fragment, probably through competition with DRN in binding to REV. The reduced *ZPR3* expression in leaf axils of P_10_ and older leaves (Fig. 7A, Fig. S8D-H) correlates with, and can explain, the enhanced DRN-REV interaction in old leaf axil-enriched tissues (Fig. 6A). The strong DRN-REV interaction in mature leaves then explains the observed upregulation of *STM* expression beginning in P_11_ (Shi et al., 2016), immediately prior to the morphological appearance of axillary buds. In fact, overexpression of *ZPR3* leads to AM initiation defects (Fig. S8I-K), which is consistent with a previous report (Kim et al., 2008) and further confirms that reduced *ZPR3* expression in mature leaf axils is crucial for the upregulation of *STM* expression and subsequent AM initiation.

## DISCUSSION

Shoot branching is fundamental to the radiation of plants, and is a key determinant of plant architecture (Coudert et al., 2015). In seed plants, shoot branching results from the lateral initiation of AMs and subsequently buds, which can become dormant until they perceive permissive environmental or internal cues to allow bud outgrowth and, thus, fine tune development. Although the outgrowth of axillary buds has been well studied, their initiation remains less well understood. Recent studies have shown that specification is an early event in which a population of *STM*-expressing meristematic cells is precisely regulated to initiate AMs (Greb et al., 2003; Long and Barton, 2000; Shi et al., 2016). Although a low level of *STM* expression maintains meristematic competence, a high level of expression leads to meristem initiation, suggesting a threshold model (Shi et al., 2016). The fine tuning of *STM* expression is therefore crucial for AM initiation.

In this study, we identified DRN and DRNL as redundant regulators of AM initiation (Fig. 1). Robust axillary bud formation in wild-type plants relies on *DRN* and *DRNL* functions. These two related AP2-family transcription factors have highly similar expression patterns in leaf axils (Fig. 2). Furthermore, DRN and DRNL directly promote *STM* expression by binding to its promoter (Figs 3-5), which not only explains the roles of DRN/DRNL in AM initiation, but also their overexpression functions. The ectopic activation of *STM* can at least partially explain the observed ectopic shoot regeneration, and the enlarged and disorganized SAM phenotypes following the constitutive overexpression of DRN or DRNL or in the *drn-D* mutant (Banno et al., 2001; Ikeda et al., 2006; Kirch et al., 2003). The transcription of *STM* is also regulated by additional unrelated *cis*-elements in its promoter (Aguilar-Martínez et al., 2015; Uchida et al., 2007). These *cis*-elements restrict *STM* expression, and are expected to function together with the *cis*-elements identified in this study to facilitate spatiotemporal *STM* expression. We and others have also shown that DRN and DRNL directly activate the expression of *CUC* genes (Ikeda et al., 2006; Matsuo et al., 2009; Tian et al., 2014), which might indirectly regulate *STM* expression and affect AM initiation (Hibara et al., 2006; Raman et al., 2008). How these genes interact temporally and spatially during AM development, especially at reproductive stages, requires further study.

**Fig. 8.**
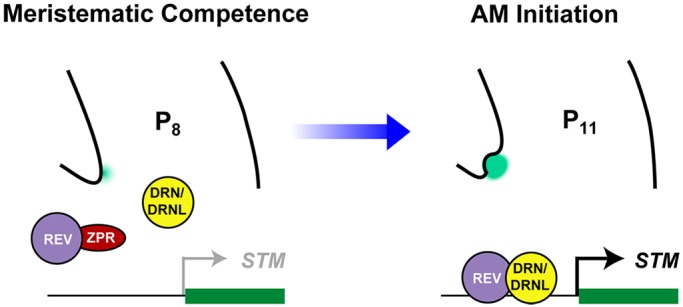
**A model summarizing the upregulation of *STM* expression prior to AM initiation through the licensing of active DRN/DRNL-REV complexes by ZPRs.** In the early leaf axil, ZPR3 is expressed and interacts with REV to inhibit the formation of functional DRN/DRNL-REV complexes, resulting in a low level of STM expression and the absence of axillary bud formation. As the leaf matures, decreasing ZPR3 levels allow the formation of DRN/DRNL-REV complexes in more mature leaf axils to upregulate STM expression and to promote axillary bud formation.

More importantly, our results demonstrate that a novel combination of interacting transcriptional regulators form a regulatory circuit that controls AM initiation during leaf maturation. We have provided *in planta* evidence that DRN/DRNL and REV form a protein complex in the leaf axils that binds to the *STM* promoter to fully upregulate its expression (Fig. 6). In addition, ZPR3 competes with DRN/DRNL for access to REV and thereby titrates the DRN/DRNL-REV interaction (Fig. 7). Thus, the balance between ZPR3 and DRN/DRNL appears to be important for DRN/DRNL-REV complex formation and *STM* expression. Indeed, temporal changes in *DRN*, *DRNL* and *ZPR3* expression can be monitored in the leaf axil (Figs 2 and 7). Early in leaf primordium development, ZPR3 is expressed in the leaf axil, where it might inhibit the formation of functional DRN/DRNL-REV complexes, resulting in a low level of *STM* expression and the absence of axillary bud formation. As the leaf matures, decreasing ZPR3 levels (Fig. 7A) would allow the formation of DRN/DRNL-REV complexes in more-mature leaf axils (Fig. 6) to upregulate *STM* expression and to promote axillary bud formation (Fig. 8). In an opposing manner, DRN and DRNL transcription is upregulated in the axils of leaves as they mature (Fig. 2). Hence, the competing interaction between DRN/DRNL and ZPR3 for REV, as well as their dynamic expression, provides a very plausible scenario to explain the observed axillary bud formation process (Greb et al., 2003; Long and Barton, 2000). Further confirmation of the ZPR3-DRN/DRNL interaction in developmental processes requires further experiments, such as manipulating the temporal and spatial expression patterns of these proteins. However, our findings underscore the importance of protein-protein interactions as a recurring feature in transcriptional regulatory networks (Brady et al., 2011).

This study has revealed that the DRN/DRNL-REV module, which is involved in embryogenesis (Chandler et al., 2007), and the ZPR–REV module, which is involved in leaf polarity patterning (Kim et al., 2008; Wenkel et al., 2007), are recruited and combinatorially regulate AM initiation. Although AM initiation, embryogenesis and leaf polarity patterning are clearly distinct developmental processes, they appear to involve conserved regulatory modules. Modifications of ancestral regulatory modules to create novel expression domains may lead to new combinations of regulatory modules and thus to regulatory neofunctionalization (Rosin and Kramer, 2009).

## MATERIALS AND METHODS

### Plant materials and generation of transgenic plants

*Arabidopsis thaliana* ecotypes Col-0 and L*er* were used as wild-type controls. The *drn-1*, *rev-6*, *pREV::REV-GR-HA*, *pDRN::GUS*, *pDRNL::GUS*, *pZPR3::GUS*, *p35S::ZPR3*, *pDRN::DRN-GFP*, *pDRN::erGFP* and *pDRNL::DRNL-CFP* lines are in the Col-0 background (Chandler et al., 2011; Cole et al., 2009; Kirch et al., 2003; Nag et al., 2007; Otsuga et al., 2001; Wenkel et al., 2007), and the *drnl-1*, *drnl-2*, *pREV::REV-Venus* and *pSTM::STM-Venus* lines are in the L*er* background (Chandler et al., 2007; Heisler et al., 2005; Nag et al., 2007). Both *drn-1* and *drnl-1* are insertion mutants. The position of the insertion in the *drn-1* allele is after nucleotide +327 (relative to the ATG) of the *DRN* gene and for *drnl-1* it is after nucleotide +777. The *drnl-2* allele has a base substitution from C to T at position +278, resulting in an A to V substitution at amino acid 93. The *rev-6* allele has an premature stop codon after R346. Plants were grown in the greenhouse on soil at 22°C under short-day conditions (8 h light/16 h dark) for 28 to 30 days and were induced to flower under long-day conditions (16 h light/8 h dark) for 30 days unless otherwise specified.

The *p35S::DRN-GR* construct was created by inserting the *DRN*-coding sequence amplified from cDNA in-frame upstream of the GR-coding sequence in the *pGREEN0229-35S::GR* vector; the T3 generation of homozygous plants of *p35S::DRN-GR* and *pREV::REV-GR-HA* were used for genotyping; *pSTM::GUS* was constructed using an amplified 6.3 kb fragment upstream of the *STM*-coding region. To construct *pSTMΔ::GUS*, region 1 (Fig. 5A) was removed, to result in a 6.0 kb fragment. All constructs were transformed into Col-0 plants using the *Agrobacterium*-mediated floral dip method. Multiple transgenic lines (>20 for each construct) were obtained, and lines with representative phenotypes or expression patterns were used for analysis.

### Hormone treatment and RT-PCR

For Dex treatment, a 10 mM stock solution of Dex (Sigma-Aldrich) in ethanol was diluted with distilled water to a final concentration of 10 μM. Water with only ethanol was added to leaf axils as the mock control. For expression analyses, plants were grown for 21 days under short-day conditions and meristematic and boundary tissue was enriched by the manual dissection of leaves from the shoot apex. Total RNA was extracted using the AxyPrep Multisource RNA Miniprep kit (Corning). First-strand cDNA synthesis was performed with 2 μg total RNA using TransScript One-Step gDNA Removal and cDNA synthesis SuperMix (TransGen), and 22-mer oligo dT primers according to the manufacturer's instructions. The RT-PCR analysis was performed in a 20 μl reaction volume using Taq DNA polymerase (TianGen) and gene-specific primers (Table S1). Quantitative RT-PCR (RT-qPCR) was performed on a Bio-Rad CFX96 real-time PCR detection system with a KAPA SYBR FAST qPCR kit (KAPA Biosystems). Relative RT-qPCR expression was normalized to that of *TUB6* (*At5g12250*), which has been shown to be a superior reference gene for RT-qPCR analysis and shows constant expression after various treatments (Kaufmann et al., 2010; Han et al., 2014; Tian et al., 2014). The relative expression level of the positive control was transformed to a value of 1 and was double-normalized by the expression of the reference gene and by the ratio of the positive control. Data for qPCR are -ΔΔCt ±s.d. of three biological replicates, each performed in triplicate. Gene-specific primers (Table S1) were used to amplify and detect each gene.

### Tissue preparation, confocal analysis and scanning electron microscopy

Seedlings were grown in MS medium in short-day conditions (8 h light at 22°C and 16 h dark at 18°C) for 15 days after seed stratification. Leaves between P_5_ and P_11_ were then detached from seedlings, laid flat on MS medium and imaged. For sectioning, seedlings were grown in soil in short-day conditions for 21 days. Shoot apices were collected, the leaves were removed and the apices were immediately placed in 2.5% paraformaldehyde (PFA; Sigma-Aldrich) at pH 7.0 at 4°C, and were then vacuum infiltrated for 30 min and stored overnight at 4°C. Fixed tissue samples were washed with 10% sucrose and 1% PFA at pH 7.0 for 20 min, with 20% sucrose and 1% PFA at pH 7.0 for 20 min, and 30% sucrose and 1% PFA at pH 7.0 for 30 min. Samples were then embedded in 5-7% LM agarose (Promega) liquid gel at 30°C and placed at 4°C for 15 min to solidify. Sections of 40-70 µm were prepared using a Leica VT1000S vibratome. For high-resolution images, samples were stained with 50 µg/ml propidium iodide (PI, Sigma-Aldrich).

Images were taken with Nikon A1 confocal and Leica SP5 microscopes. Excitation and detection wavelengths for GFP, chlorophyll and Venus were as previously described (Wang et al., 2014a,b). To detect the signal of FM4-64 and propidium iodide staining, a 514 nm laser line was used for excitation and a 561 nm long-pass filter was used for detection. Maximum projection was used in the Nikon A1 software or LAS AF Lite software.

Scanning electron microscopy was performed using a Hitachi S-3000N variable pressure scanning electron microscope after standard tissue preparation (Wang et al., 2014b).

### Chromatin immunoprecipitation and gel-shift assay

ChIP experiments were performed according to published protocols (Kaufmann et al., 2010). Shoots without leaves of ∼28 d short-day-grown *pREV::REV-GR-HA* (induced with Dex for 4 h), *pDRN::DRN-GFP* and *pDRNL::DRNL-CFP* plants were harvested and fixed with 1% formaldehyde under vacuum for 10 min. Nuclei were isolated and lysed, and chromatin was sheared to a mean size of 1000 bp by sonication. The sonicated chromatin served as an input or as a positive control. Immunoprecipitations were performed using an antibody against the glucocorticoid receptor (GR) (PA1 516; Affinity Bioreagents) or against GFP (11814460001, Roche). The precipitated DNA was isolated, purified and used as a template for PCR. qPCR was performed as described above (see Table S1 for primers). The data are presented as the degree of enrichment of *STM* promoter fragments. The amount of precipitated DNA used in each assay was determined empirically, so that an equal amount of *ACTIN2* (At3g18780) was amplified. Three independent biological replicates were performed.

The nucleotide sequences of the double-stranded oligonucleotides for EMSA were *STM* P1. The oligonucleotides were annealed and then labeled with the Biotin 3′ End DNA Labeling Kit (Pierce). Standard reaction mixtures (20 ml) for EMSA contained 2 mg purified proteins, 2 ml biotin-labeled annealed oligonucleotides, 2 ml binding buffer [100 mM Tris, 500 mM KCl, 10 mM DTT (pH 7.5)], 1 ml 50% glycerol, 1 mLl1% NP-40, 1 ml 1 M KCl, 1 ml 100 mM MgCl_2_, 1 ml 200 mM EDTA, 1 ml 1 mg ml^−1^ poly (dI-dC) and 8 ml ultrapure water. The reactions were incubated at room temperature (25°C) for 20 min and loaded onto a 10% native polyacrylamide gel containing 45 mM Tris, 45 mM boric acid, 1 mM EDTA (pH 8.3). The gel was sandwiched and transferred to an N+ nylon membrane (Millipore) in 0.56 TBE buffer at 380 mA at 4°C for 60 min. The detection of biotin-labeled DNA chemiluminescence was carried out according to the manufacturer's instructions using a LightShift Chemiluminescent EMSA Kit (PIERCE).

### Protoplast transient expression assay

To produce the effector constructs, full-length *REV*, *DRN* and *DRNL* open reading frames were amplified from *Arabidopsis* cDNA and inserted into the pBI221 vector under control of the CaMV *35S* promoter. To generate the *pSTM::Luc* reporter gene, the *STM* promoter was amplified from *Arabidopsis* genomic DNA and PCR fragments were inserted into the corresponding sites of the YY96 vector (Zhang et al., 2012) to produce *pSTM::Luc* and *pSTMΔ::Luc* (see Table S1 for primers). The YY96 vector contained a CaMV *35S* minimal promoter before the *Luc* gene. The PEG-mediated transfection of *Arabidopsis* protoplasts was performed as described previously (Han et al., 2014; Zhang et al., 2012). The reporter construct, effector plasmid and a *p35S::GUS* construct (internal control) were co-transformed into protoplasts. After transformation, the protoplasts were incubated at 23°C for 12-15 h. The protoplasts were pelleted and resuspended in 100 μl of 1× CCLR buffer (Promega). For GUS enzymatic assays, 5 μl of extract was incubated with 50 μl 4-methylumbelliferyl-β-d-glucuronide assay buffer [50 mM sodium phosphate (pH 7.0), 1 mM β-d-glucuronide, 10 mM EDTA, 10 mM β-mercaptoethanol, 0.1% sarkosyl, 0.1% Triton X-100] at 37°C for 15 min, and the reaction was stopped by adding 945 μl 0.2 M Na_2_CO_3_. For luciferase activity assays, 5 μl of the extract was mixed with 50 μl luciferase assay substrate (Promega), and the activity was detected using a Modulus Luminometer/Fluometer (Promega) and a luminescence kit. The reporter gene expression levels were expressed as relative LUC/GUS ratios. Three independent biological experiments were each performed in triplicate.

### Co-immunoprecipitation assay

*Arabidopsis* plants expressing REV-MYC and DRN-GFP were used in a co-immunoprecipitation (IP) assay. Shoot apices of 1-week-old (younger axils than the tenth leaves) or leaf axils of 3-week-old (mature axils older than the tenth leaves) transgenic plants were ground in IP buffer [20 mM HEPES (pH 7.5), 40 mM KCl, 1 mM EDTA and 1% Triton X-100], filtered and centrifuged at 20,000 ***g*** for 10 min. Supernatants containing equal amounts of DRN-GFP were incubated with anti-GFP coupled to Protein A sepharose beads for 30 min. Beads were washed four times with wash buffer [20 mM HEPES (pH 7.5), 40 mM KCl, and 0.1% Triton X-100] and bound proteins were eluted with 2× SDS buffer.

### Yeast two-hybrid and three-hybrid assays

The yeast two-hybrid screens were performed as described previously (Zhang et al., 2012). To reduce autoactivation, 100 mM 3-AT (3-amino-1,2,4-triazole) was added to the selection medium. We detected no auto-activation of BD-REV in the presence of 3-AT. A yeast three-hybrid assay was performed according to the manufacturer's instructions; the yeast strain AH109 was transformed with pairs of plasmids (pB-REV-DRN/DRNL and pA-ZPR3 or pB-REV-DRN/DRNLΔAP and pA-ZPR3).

Transformed colonies were selected on synthetic complete medium lacking Leu and Trp. Three independent clones with four respective replicates were used in each experiment. Cell cultures were placed under the indicated light conditions and incubated at 30°C until the OD_600_ was between 0.5 and 0.8 with the conditional expression of the bridge proteins. The relative β-galactosidase activities were calculated as described previously (Ding et al., 2015). At least three independent experiments were performed and the result of one representative experiment is shown.

## Supplementary Material

Supplementary information
