# Supplementary material for: Spatiotemporal control of axillary meristem formation by interacting transcriptional regulators
Source: Development. 2018 Dec 10;145(24):dev158352. doi: 10.1242/dev.158352 (PMC6307885; doi:10.1242/dev.158352)
Supplement: Supplementary information [file develop-145-158352-s1.pdf]

**Supplemental Table 1.** Primers used in this study.

| Primer | Sequence(5'-3') |
|---|---|
| RT-PCR STM-F | GGCCGCTTATGTCAATTG (95%) |
| RT-PCR STM-R | AGTAAGCTTCCATGAATTG |
| ChIP STM-1F | AGGGTTTCCCAATAAATTTA (91%) |
| ChIP STM-1R | CCATGATGTTGATGTGATG |
| ChIP STM-2F | GAAGAACACAAAAGAGTTA (93%) |
| ChIP STM-2R | AAAAGAGTAAGTAACTGTGT |
| ChIP STM-3F | TACTTTTTAAACCAACATC (93%) |
| ChIP STM-3R | TGCTGAAATATGATTTCTC |
| ChIP STM-4F | TATTTATGACATTTTTCTTC (99%) |
| ChIP STM-4R | CATTTGTGTATATGTATATA |
| ChIP STM-5F | AGGTACAGGTGTCCCATGCT (98%) |
| ChIP STM-5R | TAATCATAGAGTAAAACATGT |
| ChIP STM-6F | TAGTGATAACAAAATTAGC (92%) |
| ChIP STM-6R | GTTTATCCCTGAAAATAGT |
| ChIP STM-7F | AGTTTCTCAGTTATCTTTTC (96%) |
| ChIP STM-7R | TTCGAAATATTATTTAGAAG |
| ChIP STM-8F | AAATATTTATATCTAATAT (96%) |
| ChIP STM-8R | AATAGATAGAGAGAGATTA |
| ChIP ACTIN-F | GTCGTACAACCGGTATTGTGC (99%) |
| ChIP ACTIN-R | CACAAACGAGGGCTGGAACAAG |
| pSTM-F | *AACTGCAG*AGACGTAGTAATACTAGA |
| pSTM-R | *GCTCTAGA*CTACTTTGTTGGTGGTGTG |
| pSTM*Δ*-R | *GCTCTAGA*GAAACACATTAAAGTACTA |
| RT-PCR ZPR1-F | GTTCTTCAGAAACATTTTC (98) |
| RT-PCR ZPR1-R | CTCCACGTAAAGCTTTAG |
| RT-PCR ZPR3-F | ACTGTTACATAATGAAAG (96%) |
| RT-PCR ZPR3-R | AGATTGTCCAGAAGCAGA |
| ChIP ZPR1-F | CTTTAAACGAATAATGCAA (93%) |
| ChIP ZPR1-R | AGCTCCAAGATGTGTTTAA |
| DRN F1 | GAAACTTCACCGAATCTACCC |
| DRN R1 | GCCGCAGAGTCGTAAGCACAA |
| DRN R2 | GAGCCGTTGAAAGAGTTATT |
| DRNL F1 | CGAAGGTGCCGAGCACAGA |
| DRNL F2 | TACCGCAAAAGCTGCCTC |
| DRNL R1 | TCCACCATTTCCGTTCTGC |
| DRNL R2 | AGCGGCGCAGTCATATGCGCAGTCT |
| GR F1 | GAGGTGATTGAACCCGAGG |
| GR F2 | ACCGTTGCCAGTTCTGAC |

The values in brackets indicate the efficiency of qPCR primers.

Development • Supplementary information

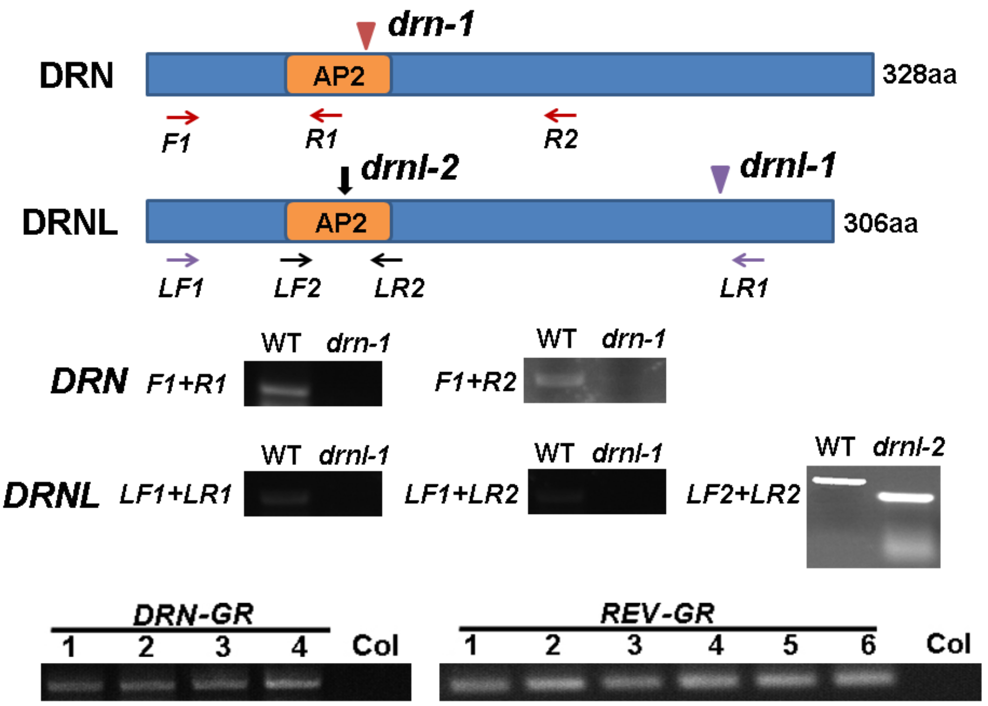


**Fig. S1.** Schematic diagrams of DRN and DRNL proteins showing the positions of mutations in the corresponding encoding genes.

The red arrow at position 107 indicates the insertion site of the *dSpm* element into the AP2 domain-encoding sequence in the *drn-1* allele (Kirch et al., 2003). F1+R1 and F1+R2 could not amply bands from cDNA of *drn-1* mutant, indicating that *drn-1* is a null allele. In *drnl-1*, transposon-flanking DNA was inserted at position 259 near C terminal. LF1+LR1 and LF1+LR2 could not amplify bands from cDNA of *drnl-1*. In *drnl-2* arrow indicates the single nucleotide substitution that results in an A to V substitution at position 93 in the AP2 domain. Homozygosity was confirmed using a dCAPS marker with primers LF2 and LR2, which give a wildtype amplicon of 184 bp that is cleaved in the mutant by *Acc*I into two fragments of 159 and 25 bp. Different

Development • Supplementary information

*p35S::DRN-GR* and *pREV::REV-GR-HA* transgenic lines (labeled with a number) were genotyped using genomic DNA and primers for *GR.* Line1 for each transgenic line was used in the experiments, except where otherwise indicated.

Development • Supplementary information

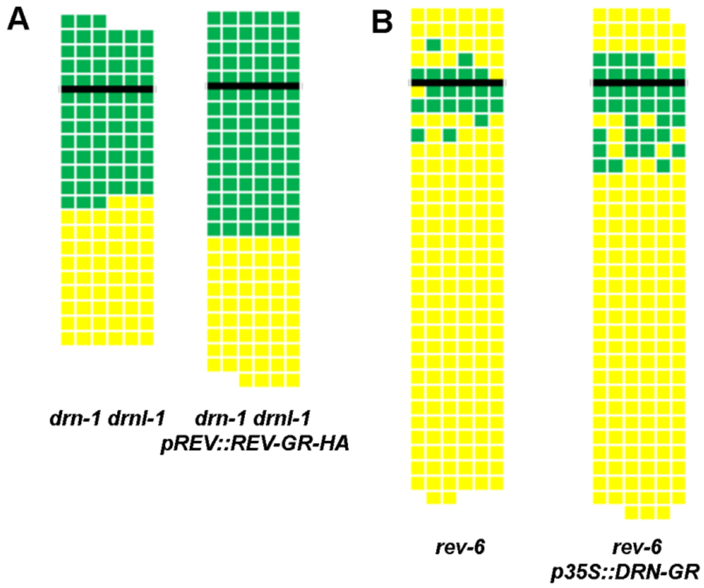


**Fig. S2.** Frequency of axillary buds in *pREV::REV-GR-HA drn-1 drnl-1*and *p35S::DRN-GR rev-6*.

The number of axillary buds was not affected in early rosette leaves but slightly increased in the axil of later rosette leaves and cauline leaves following Dex treatment in *drn-1 drnl-1 pREV::REV-GR-HA* plants (A) and *rev-6 p35S::DRN-GR* plants (B) compared with the controls. The thick black horizontal line represents the border between the youngest rosette leaf and the oldest cauline leaf. Each column represents a single plant, and each square within a column represents an individual leaf axil. The bottom row represents the oldest rosette leaf axils, with progressively younger leaves above. Green indicates the presence of an axillary bud, and yellow indicates the absence of an axillary bud in any particular leaf axil.

Development • Supplementary information

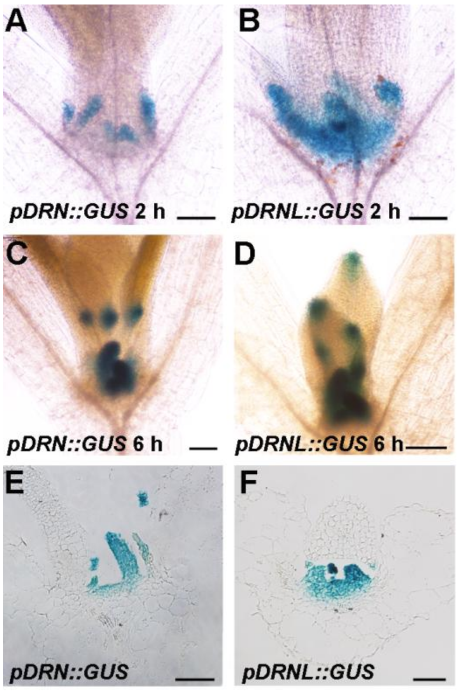


**Fig. S3.** *DRN* and *DRNL* expression patterns in shoot apices and young leaf primordia.

(A) and (C) Expression pattern of *pDRN::GUS* in the vegetative shoot apex and leaf primordia of 10-d-old plants. Seedlings were stained for 2 h in (A) and 6 h in (C).

(B) and (D) Expression pattern of *pDRNL::GUS* in the vegetative shoot apex and leaf primordia of 10-d-old plants. Seedlings were stained for 2 h in (B) and 6 h in (D).

(E) and (F) Longitudinal sections for *pDRN::GUS* (E) and *pDRNL::GUS* (F), showing expression in the shoot apex. Material was stained for 2 h.

Bars = 100 µm in (A) to (D), and 50 µm in (E) and (F).

Development • Supplementary information

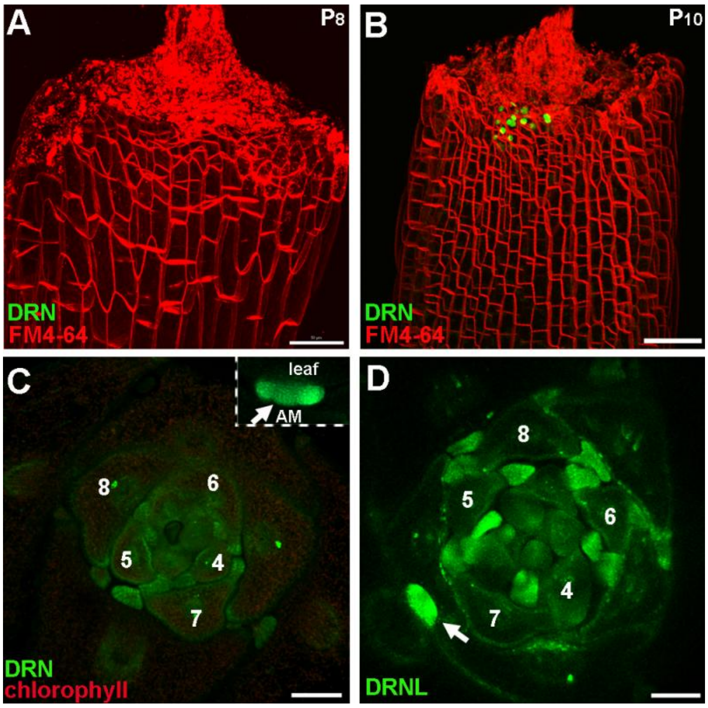


**Fig. S4.** *DRN* is expressed prior to AM initiation in leaf axils.

(A) and (B) Reconstructed view of the epidermal layer of a $P_8$-stage (A) and a $P_{10}$-stage (B) leaf axil with *pDRN::DRN-GFP* expression in green and FM4-64 stain in red showing the location of AM progenitor cells. Both leaves were excised from 17-d-old plants. Note the enrichment of *DRN-GFP* signals in the $P_{10}$-stage leaf axil.

(C) and (D) Transverse sections of *pDRN::DRN-GFP* and *pDRNL::DRNL-CFP* plants to show the decreased level of *DRN* and *DRNL* expression in mature leaves (>$P_8$) and strong expression in the axillary meristem (arrows). The seedling was 21-days-old and the leaf showing an axillary meristem highlighted in the inset in (C) was $P_{15}$.

Bars = 50 µm.

Development • Supplementary information

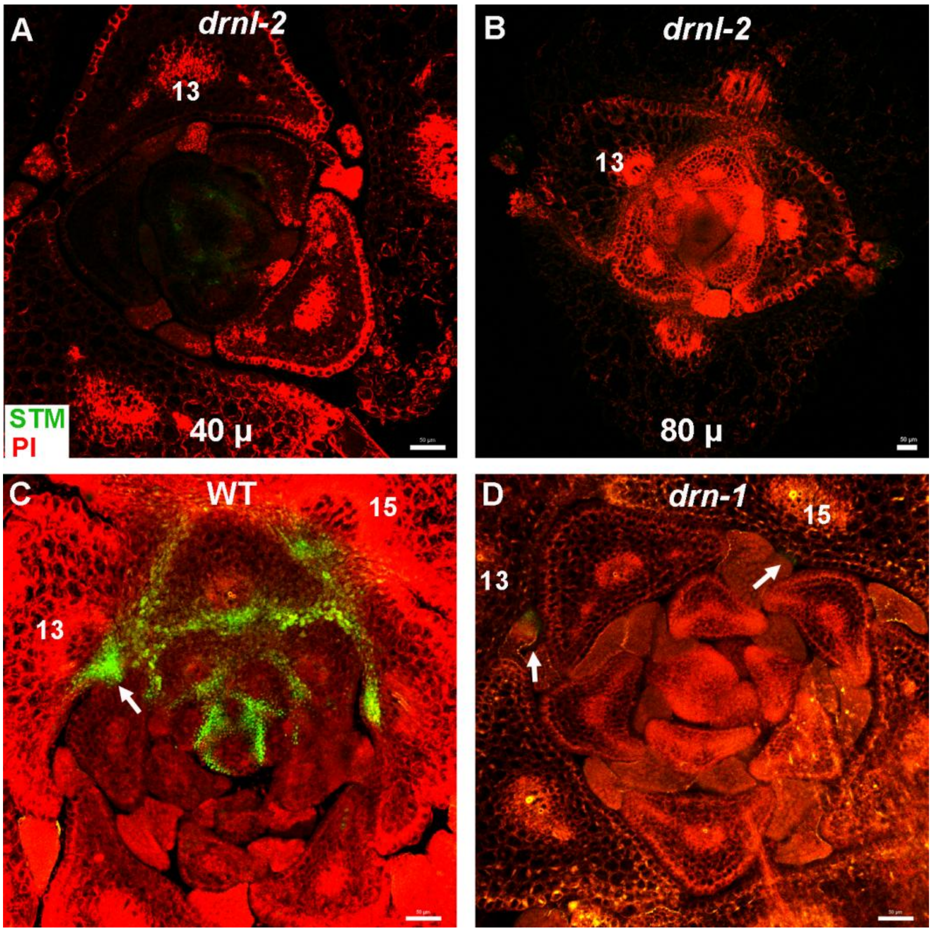


**Fig. S5.** The expression pattern of *pSTM::STM-Venus* in *drnl-2* and *drn-1* mutants.

(A) and (B) *STM* was weakly expressed in young and mature leaves. Images were obtained from continuous sections from one *drnl-2* plant.

(C) and (D) *STM-Venus* signals were strong in mature leaves of wild-type (arrow represents $P_{13}$), but were severely attenuated in *drn-1* mutants (arrows represent $P_{13}$ and $P_{15}$). Sections for wild-type and *drn-1* were at an equivalent position.

Bars = 50 μm.

Development • Supplementary information

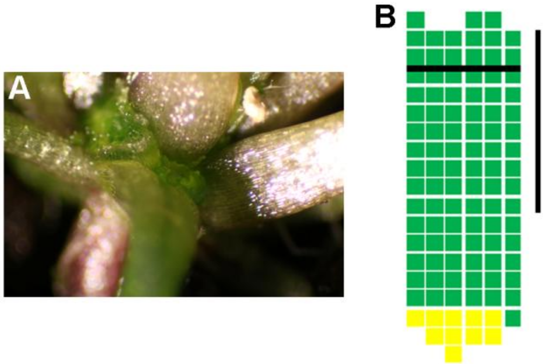


**Fig. S6.** *STM* overexpression complemented the reduction in axillary meristems in *drn-1 drnl-1 rev-6* mutants.

(A) Dex induced axillary meristems in *p35S::STM-GR drn-1 drnl-1 rev-6* plants.

(B) The number of axillary buds increased in *p35S::STM-GR drn-1 drnl-1 rev-6* plants after Dex treatment. Plants were grown in short-days for 15 d without treatment; leaf axil regions were treated with 10 µM Dex every second day for another 15 d and then shifted to long-day conditions without treatment until axillary buds were counted. The vertical line indicates leaves initiated during Dex treatment.

The thick black horizontal line represents the border between the youngest rosette leaf and the oldest cauline leaf. Each column represents a single plant, and each square within a column represents an individual leaf axil. The bottom row represents the oldest rosette leaf axils, with progressively younger leaves above. Green indicates the presence of an axillary bud, and yellow indicates the absence of an axillary bud in any particular leaf axil.

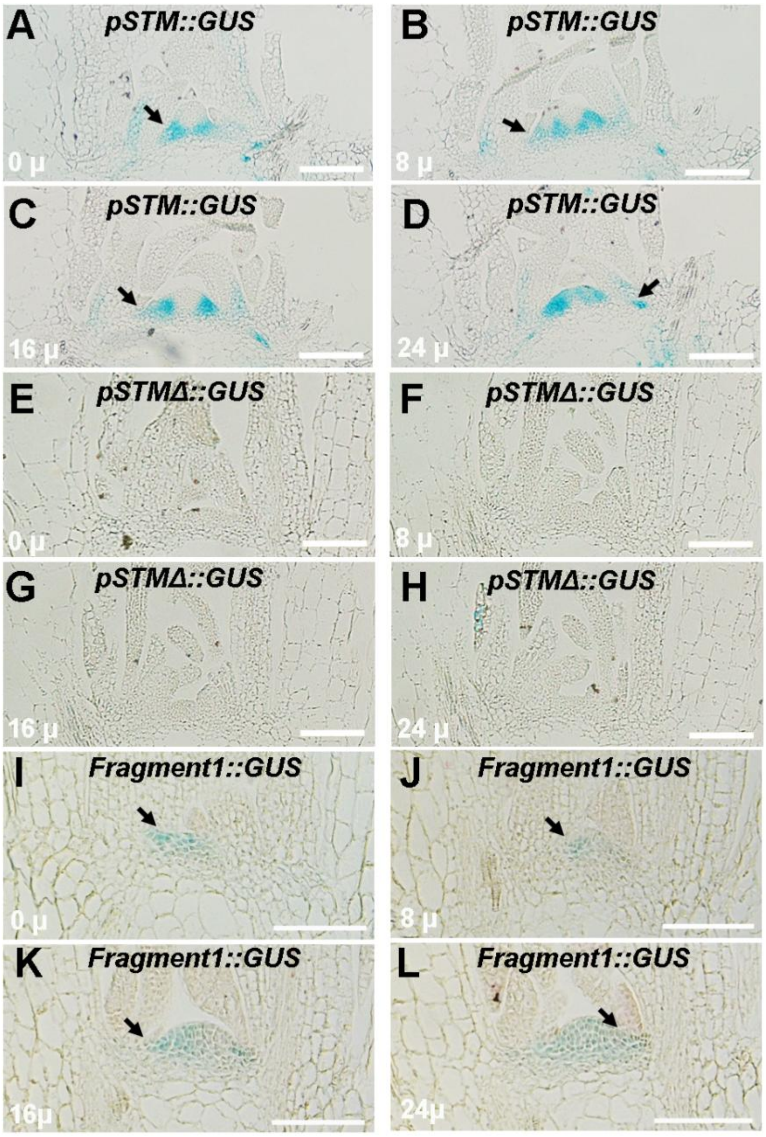


**Fig. S7.** The expression patterns of *pSTM::GUS*, *pSTMΔ::GUS* and *fragment1::GUS*.

Serial longitudinal 8-μm thick sections through vegetative shoot apices of 30-d-old plants showing the pattern of GUS expression driven by *pSTM* (A–D), *pSTMΔ* (E–H) or *fragment1::GUS* (I–L). To compare the signals, the plants were stained under the same conditions. Note the reduction in GUS expression in the leaf axils of *pSTMΔ::GUS* plants and the weak GUS expression in *fragment1::GUS* plants. Arrows highlight leaf axils. Bars = 100 μm.

Development • Supplementary information

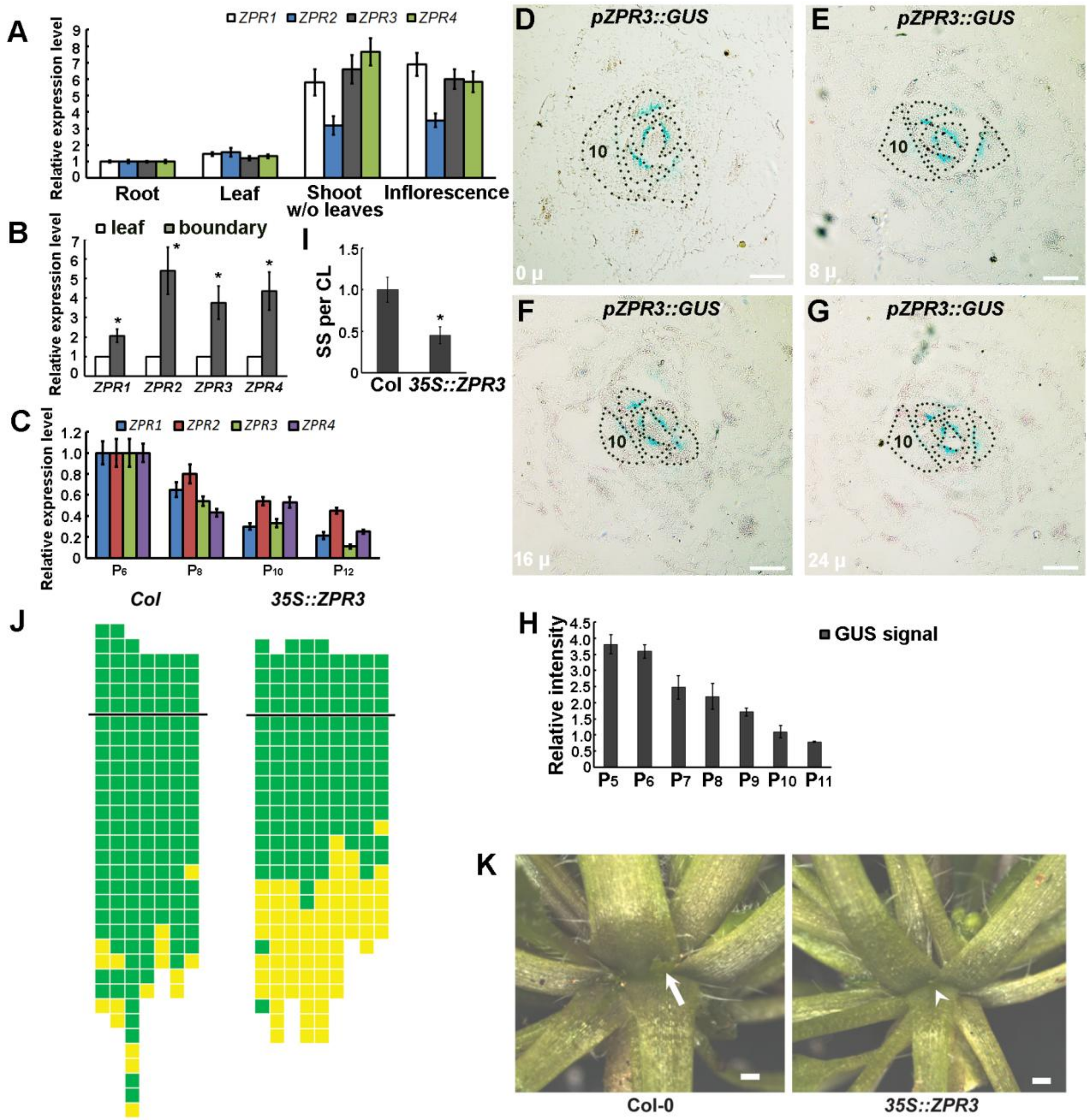


**Fig. S8.** The expression pattern of *ZPR* in leaf axils.

(A) RT-qPCR analysis of *ZPR1*, *ZPR2, ZPR3* and *ZPR4* expression levels in roots, leaves, shoots without leaves, and inflorescences. The latter two tissues are enriched with boundary tissues following leaf removal. Error bars indicate the SD.

(B) RT-qPCR analysis of *ZPR* expression in leaves and shoot apices without

Development • Supplementary information

leaves, which are enriched with boundary tissues. Error bars indicate the SD. *$P < 0.01$, (Student's *t*-test).

(C) RT-qPCR analysis of *ZPR* gene expression at different leaf stages.

(D) to (G) Pattern of *ZPR3*-promoter-driven GUS expression in serial transverse sections through the vegetative shoot apex of a 30-d-old wild-type-like plant. The 8-μm thick sections are ordered from the most apical (D) to the most basal (G); the approximate distance from the summit of the meristem to the midpoint of the section is shown in the bottom left-hand corner of each image. Note that the GUS signals decrease in intensity in the axils of older leaves. Bars = 100 μm.

(H) Measurement of GUS intensity in different leaves of 10, *pZPR3::GUS* plants.

(I) Frequency of secondary shoots per cauline leaf in wild-type and *p35S::ZPR3* transgenic lines. *$P < 0.01$, (Student's *t*-test).

(J) Schematic of the frequency of axillary buds in *p35S::ZPR3* plants. Green indicates the presence and yellow indicates the absence of an axillary bud in any particular leaf axil.

(K) Close up of views of an exemplary *p35S::ZPR3* transgenic plant showing an axillary meristem defect compared to wild type. Bars = 100 μm.

Development • Supplementary information

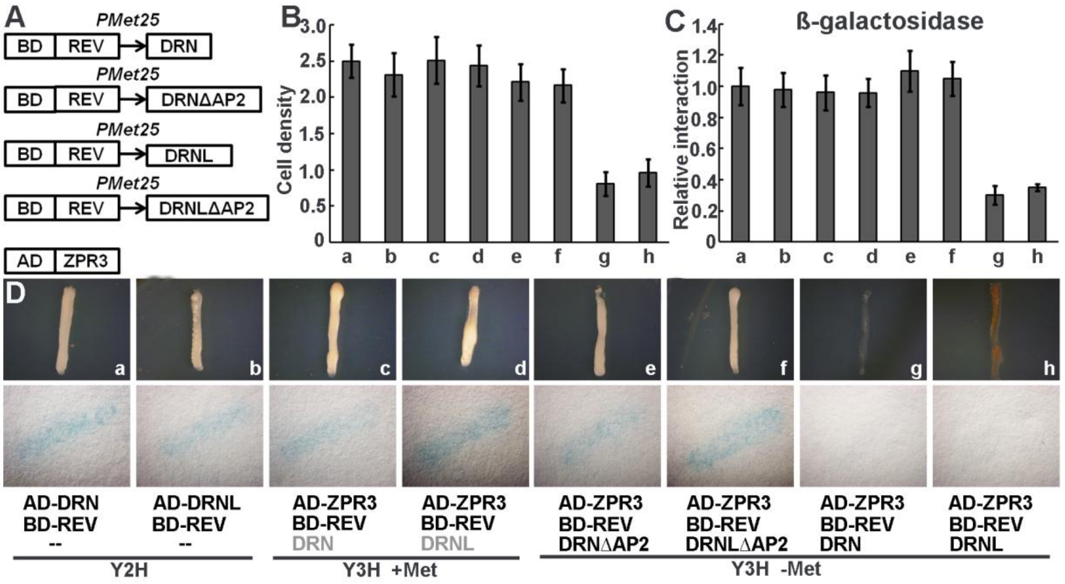


**Fig. S9.** ZPR3 interferes with the DRN/DRNL–REV interaction.

(A) A schematic representation of the constructs for Y3H vectors. DRN/DRNL and DRN/DRNLΔAP2 were inserted into the BD vector and driven by the *Met25* promoter, which can be suppressed by Met. In a complementary assay, DRN was replaced by ZPR3 in the BD vector.

(B) to (D) Yeast growth on SD-Leu-Trp-His-Ade plates and staining for β-galactosidase activity. Cell density quantification after incubation for 16 h (B), relative β-galactosidase activity of the transformed yeast (C), and staining for β-galactosidase activity (D) were performed. The addition of Met inhibited the activity of the *Met25* promoter.

Development • Supplementary information
